# Ultrasensitive Capture of Human Herpes Simplex Virus Genomes Directly from Clinical Samples Reveals Extraordinarily Limited Evolution in Cell Culture

**DOI:** 10.1128/mSphereDirect.00283-18

**Published:** 2018-06-13

**Authors:** Alexander L. Greninger, Pavitra Roychoudhury, Hong Xie, Amanda Casto, Anne Cent, Gregory Pepper, David M. Koelle, Meei-Li Huang, Anna Wald, Christine Johnston, Keith R. Jerome

**Affiliations:** a Department of Laboratory Medicine, University of Washington, Seattle, Washington, USA; b Fred Hutchinson Cancer Research Institute, Seattle, Washington, USA; c Department of Medicine, University of Washington, Seattle, Washington, USA; d Department of Global Health, University of Washington, Seattle, Washington, USA; e Benaroya Research Institute, Seattle, Washington, USA; f Department of Epidemiology, University of Washington, Seattle, Washington, USA; Icahn School of Medicine at Mount Sinai; University of Cambridge; University of Colorado School of Medicine

**Keywords:** HSV-1, HSV-2, capture sequencing, culture, dual-strain infection, genomics, herpesvirus, superinfection

## Abstract

Herpes simplex viruses (HSVs) are difficult to sequence due to their large DNA genome, high GC content, and the presence of repeats. To date, most HSV genomes have been recovered from culture isolates, raising concern that these genomes may not accurately represent circulating clinical strains. We report the development and validation of a DNA oligonucleotide hybridization panel to recover nearly complete HSV genomes at abundances up to 50,000-fold lower than previously reported. Using copy number information on herpesvirus and host DNA background via quantitative PCR, we developed a protocol for pooling for cost-effective recovery of more than 50 HSV-1 or HSV-2 genomes per MiSeq run. We demonstrate the ability to recover >99% of the HSV genome at >100× coverage in 72 h at viral loads that allow whole-genome recovery from latently infected ganglia. We also report a new computational pipeline for rapid HSV genome assembly and annotation. Using the above tools and a series of 17 HSV-1-positive clinical swabs sent to our laboratory for viral isolation, we show limited evolution of HSV-1 during viral isolation in human fibroblast cells compared to the original clinical samples. Our data indicate that previous studies using low-passage-number clinical isolates of herpes simplex viruses are reflective of the viral sequences present in the lesion and thus can be used in phylogenetic analyses. We also detect superinfection within a single sample with unrelated HSV-1 strains recovered from separate oral lesions in an immunosuppressed patient during a 2.5-week period, illustrating the power of direct-from-specimen sequencing of HSV.

**IMPORTANCE** Herpes simplex viruses affect more than 4 billion people across the globe, constituting a large burden of disease. Understanding the global diversity of herpes simplex viruses is important for diagnostics and therapeutics as well as cure research and tracking transmission among humans. To date, most HSV genomics has been performed on culture isolates and DNA swabs with high quantities of virus. We describe the development of wet-lab and computational tools that enable the accurate sequencing of near-complete genomes of HSV-1 and HSV-2 directly from clinical specimens at abundances >50,000-fold lower than previously sequenced and at significantly reduced cost. We use these tools to profile circulating HSV-1 strains in the community and illustrate limited changes to the viral genome during the viral isolation process. These techniques enable cost-effective, rapid sequencing of HSV-1 and HSV-2 genomes that will help enable improved detection, surveillance, and control of this human pathogen.

## Abstract (precis)

Herpes simplex viruses affect more than 4 billion people across the globe, constituting a large burden of disease. Understanding the global diversity of herpes simplex viruses is important for diagnostics and therapeutics as well as cure research and tracking transmission among humans. To date, most HSV genomics has been performed on culture isolates and DNA swabs with high quantities of virus. We describe the development of wet-lab and computational tools that enable the accurate sequencing of near-complete genomes of HSV-1 and HSV-2 directly from clinical specimens at abundances >50,000-fold lower than previously sequenced and at significantly reduced cost. We use these tools to profile circulating HSV-1 strains in the community and illustrate limited changes to the viral genome during the viral isolation process. These techniques enable cost-effective, rapid sequencing of HSV-1 and HSV-2 genomes that will help enable improved detection, surveillance, and control of this human pathogen.

## INTRODUCTION

Herpes simplex virus 1 (HSV-1) and herpes simplex virus 2 (HSV-2) are alphaherpesviruses causing over 4 billion human infections that can manifest as oral and genital ulcerations, neonatal disease, herpetic keratitis, and encephalitis (1, 2). While HSV-2 has traditionally been associated with genital herpes, HSV-1 comprises the majority of first-episode genital herpes infections in high-income countries (3). HSV genome evolution is notable for extensive HSV-1 recombination within HSV-2 genomes, with no detectable HSV-2 recombination into HSV-1 genomes (4, 5).

To date, most human herpes simplex virus genome sequencing has been performed on culture isolates (6–10). Culture is a pragmatic method to enrich for viral sequences, and many clinical virology labs have rich banks of cultured HSV isolates. However, without the ability to compare these sequences to sequence recovered directly from clinical samples, interpretation of sequence results has been tempered by the concern that culture isolates might not accurately represent viral sequence *in vivo*. Other viruses such as influenza and parainfluenza viruses have shown that culture adaptation results in radically different viral sequence and receptor binding properties that do not accurately reflect selection pressures *in vivo* (11–13). Culture of the polyomaviruses BK and JC viruses (BKV and JCV, respectively) is often performed in simian virus 40 (SV40) large-T antigen immortalized cell lines, allowing near-complete loss of the BKV and JCV large-T antigen via transcomplementation, representing loss of one-third of the viral genome (14, 15). Culture adaptation of human herpesvirus 6A/B results in large tandem repeats in the origin of replication and other regions that are not found in low-passage-number clinical isolates and likely helps to accelerate viral replication *in vitro* (16–18). Similarly, laboratory passage of human cytomegalovirus (CMV), Epstein-Barr virus, and varicella-zoster virus can result in surprisingly large deletions comprising multiple genes and kilobases (19–22).

Many clinical studies of HSV conducted at our institution and throughout the world have utilized swabs to obtain DNA, and such samples have the advantages of being easily collected, stable at room temperature, and able to be sequenced directly from the patient. To fully take advantage of the rapidly growing field of genomics to understand HSV pathogenesis and diversity, we created a high-throughput method for sequencing HSV from DNA swab and culture material. Capture sequencing has become commonly used in human exome sequencing and oncology panels and for other herpesviruses (23–25). We report here the development of wet-lab and dry-lab tools for sequencing of HSV-1 and HSV-2 genomes directly from clinical specimens using a custom oligonucleotide hybridization panel. In our hands, these methods extended the range of HSV-1 and HSV-2 viral abundances from which whole-genome recovery is possible by up to 5 logarithms. By recovering HSV-1 sequence directly from clinical specimens, we compare sequences from HSV-1 in clinical samples with clinical isolates recovered from culture on human fibroblast cells. We show extraordinarily limited evolution of HSV-1 genomes during viral isolation. As an example of the power of our approach, we also report the first genomic detection of HSV-1 superinfection from a single oral swab.

## RESULTS

### Development of standard operating procedure for HSV genome capture.

**FIG 1:**
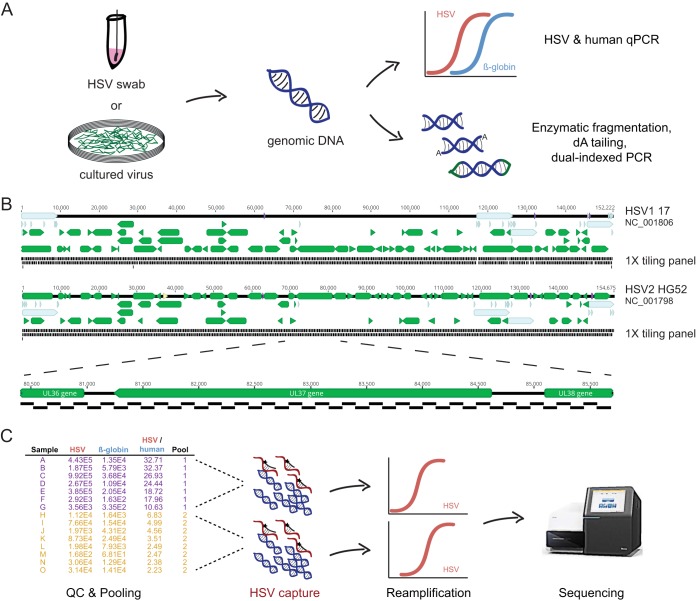
Experimental protocol. (A) DNA is extracted from either clinical swabs in proteinase K buffer or cell culture supernatant. DNA is quantitated for HSV and beta-globin; it is enzymatically fragmented, end repaired, and dA tailed; and TruSeq Y-adapters are ligated on. (B) Design of 1- by 120-bp tiling panel across HSV-1 and HSV-2 genomes. (C) Samples are pooled in sets of 4 to 10 based on the HSV/beta-globin ratio to minimize variance in viral concentration and readjusted based on the total number of HSV copies present in each sample.

To recover whole genomes directly from clinical swabs, we designed a specialized capture sequencing workflow for clinical HSV genomics. DNA is extracted from clinical swabs collected in universal transport medium or proteinase K buffer, and total DNA is quantitated (Fig. 1A). HSV and beta-globin copy number are quantitated using specific quantitative PCR (qPCR).

Based on our experience with the limited sensitivity of shotgun sequencing directly from HSV-2 clinical swabs, we developed a custom tiling oligonucleotide panel for HSV-2 based on the HG52 reference genome (NC_001798) (Fig. 1B) (9). Experiments showed that while the HSV-2 capture panel could readily recover near-complete genomes from HSV-2 material, it could recover only less than 30% of the HSV-1 genome from HSV-1 culture specimens (see Fig. S1A in the supplemental material). Recovered regions of HSV-1 correlated with its average pairwise identity to HSV-2, requiring >85% pairwise identity for high coverage (Fig. S1B). We thus designed an additional HSV-1 capture panel for subsequent HSV-1 capture experiments (Fig. 1B).

10.1128/mSphereDirect.00283-18.1 FIG S1  HSV-1 culture isolate captured with HSV-2 capture panel. Early in development, we attempted capture of an HSV-1 culture isolate with an HSV-2 capture panel. (A) Coverage map of reads across the HSV-1 genome shows that coverage was poor. Despite an average coverage of 179×, only 58% of the HSV-1 UL region had a depth of ≥10×. The *y* axis shows read depth, while the *x* axis is the genome position for HSV-1. HSV-1 genes are denoted in green, while repeat regions are highlighted in light blue. (B) HSV-1 UL locus depth correlates with pairwise identity to HSV-2 UL sequence. We calculated the pairwise HSV-1 versus HSV-2 sequence identity across a 120-nucleotide sliding window and plotted the data as a histogram (blue). For each 120-nucleotide bin across the HSV-1 UL, we calculated the median bin depth from the capture sequencing normalized to the maximum bin depth (black dots). Download FIG S1, EPS file, 1.3 MB. Copyright © 2018 Greninger et al. 2018 Greninger et al. This content is distributed under the terms of the Creative Commons Attribution 4.0 International license.

To increase the cost-effectiveness of capture sequencing, we developed a pooling scheme for performing capture on dual-indexed libraries. While pooling schemes are common in many capture sequencing protocols, dealing with potential billionfold differences in copy number between different HSV specimens along with differences in host background and variance of quantitation by qPCR required a different approach (Fig. 1C). For example, inclusion of a high-HSV-copy-number specimen in the same pool with a low-copy-number specimen could result in few reads being obtained for the lower-copy-number specimen, thus requiring reenrichment of the low-copy-number library. Our protocol ranks libraries by the relative amounts of HSV and beta-globin present, and pools of 5 to 10 specimens are chosen based on the variance of HSV/human ratio present in the samples prepared. We generally prepare 30 to 50 precapture libraries in batch, resulting in approximately 4 to 7 pools for capture. Samples in pools may be subsequently reassigned to a different pool based on the total copy number of HSV-1 and HSV-2 present. Because different amounts of HSV are present in each pool, we perform the postenrichment amplification step with an initial 10 cycles of PCR followed by monitoring of additional cycles on lower-concentration pools by either Sybr green or iterative checking by agarose gel electrophoresis to a maximum of 20 PCR cycles after capture. Pools are sequenced on 2- by 300-bp Illumina MiSeq runs to enhance recovery of particularly high-GC regions and the multiple repeats present in HSV genomes.

### Development of a custom pipeline for HSV assembly and annotation.

**FIG 2:**
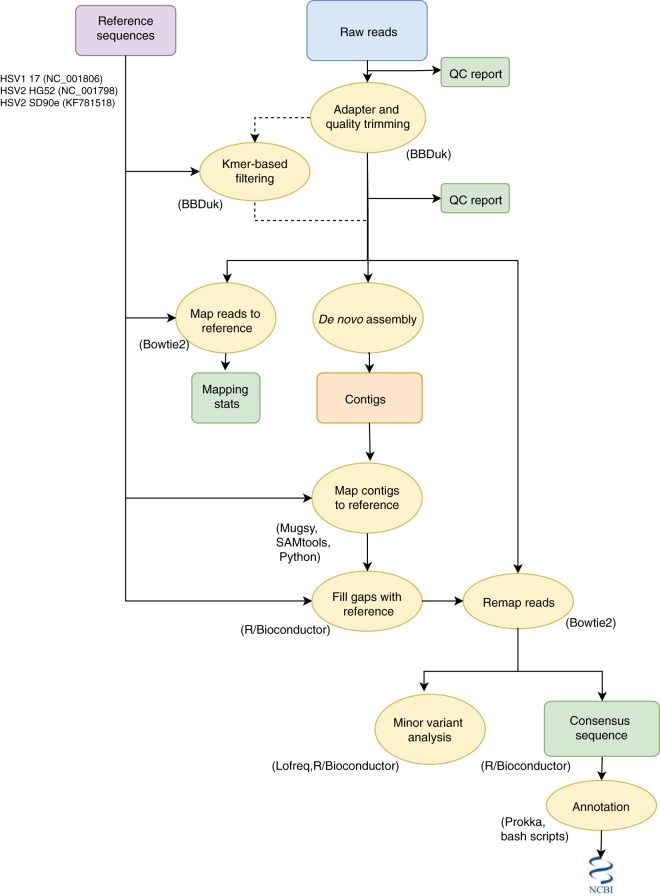
Overview of pipeline for assembly and annotation of HSV sequences. Raw reads are adapter and quality trimmed using BBDuk. If precapture shotgun HSV libraries are sequenced, trimmed reads are subjected to k-mer filtering prior to assembly to prevent tedious assembly of the human genome. Reads are *de novo* assembled using SPAdes v3.11 and mapped to each of three reference genomes to determine whether HSV-1 or HSV-2 was sequenced. Contigs are mapped to the chosen reference, and gaps are filled with reference sequence. Finally, reads are mapped to this sequence in order to determine the consensus sequence before annotation and submission to NCBI.

We developed a computational pipeline (Fig. 2) to rapidly extract and annotate near-full-length HSV genomes from raw Illumina sequencing reads. By employing a combination of reference-guided and assembly-based methods to construct consensus sequences, we were able to recover up to 99% of the genome.

The workflow starts with quality analysis of raw reads followed by trimming to remove adapters and low-quality regions. For samples sequenced without target capture enrichment or with a low percentage of HSV reads, a k-mer-based filtering method is used to enrich for HSV reads based on similarity to the HSV-1 strain 17 and HSV-2 strain HG52 and SD90e reference sequences (Fig. 2). The removal of off-target reads significantly speeds up downstream processing steps by preventing *de novo* assembly of mammalian genomes. Preprocessed reads are *de novo* assembled into contigs, and the reference sequence is used to order these contigs and fill in any gaps. Reads are then mapped to this resulting template, and custom scripts are used to construct the final consensus sequence. Finally, the consensus sequence is annotated and prepared for GenBank deposit. Our pipeline combines several previously published open-source tools with custom scripts and can be run on desktop computers, servers, and high-throughput computing clusters. On average, a single sample containing about 700,000 raw reads run on a machine with 14 cores takes about 15 min.

### Accuracy of capture-based sequencing.

To validate the accuracy of our sequencing method, we compared thymidine kinase (UL23) sequences obtained from PCR-Sanger sequencing and those obtained from our capture sequencing method for eight strains of HSV-1 and eight strains of HSV-2 (Fig. S2). For Sanger sequencing, UL23 was PCR amplified from genomic DNA and Sanger sequenced to a minimum of 2× coverage. For the whole-genome sequencing (WGS) genes, majority consensus sequence for the UL23 coding sequence (CDS) was extracted from the annotated assembly and aligned against the corresponding Sanger sequence. No consensus variants were recovered from either of the two genes in either HSV-1 or HSV-2, yielding an accuracy of 100%.

10.1128/mSphereDirect.00283-18.2 FIG S2  HSV-1 (A) and HSV-2 (B) UL23 genes show 100% identity whether sequenced by PCR-Sanger sequencing or capture panel next-generation sequencing approach. Download FIG S2, EPS file, 1.2 MB. Copyright © 2018 Greninger et al. 2018 Greninger et al. This content is distributed under the terms of the Creative Commons Attribution 4.0 International license.

### Limits of genome recovery.

**FIG 3:**
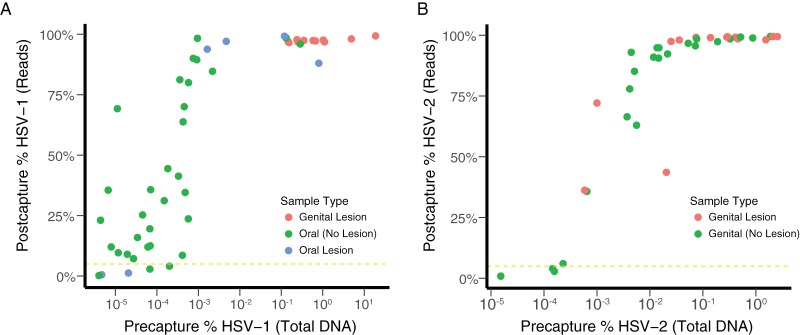
Capture sequencing allows near-complete genomes from all symptomatic HSV clinical samples. Efficiency of sequence enrichment from clinical samples for HSV-1 (A) and HSV-2 (B) is depicted. Precapture HSV percentage of total DNA is shown on the *x* axis based on qPCR values for HSV and beta-globin. Postcapture HSV percentage is shown on the *y* axis based on percentage of total reads mapping to HSV (on-target percentage). Sample types are labeled by color for genital lesion (red), oral lesion (blue), asymptomatic oral shedding (green [A]), or asymptomatic genital shedding (green [B]). The gold dashed line denotes 2% postcapture HSV reads, above which near-complete genomes were obtained.

To determine the lower limit of capture for our whole-genome sequencing method and to understand the determinants of our on-target percentage and coverage statistics, we performed capture sequencing on HSV-1 and HSV-2 clinical samples across a range of concentrations (Fig. 3; Table S1). We calculated the precapture ratio of HSV mass to human DNA mass based on the quantities of HSV and beta-globin recovered in the initial qPCRs. We then compared the precapture HSV mass ratio to the on-target fraction of HSV reads after the capture as a proxy for genome recovery.

10.1128/mSphereDirect.00283-18.3 TABLE S1  (A) HSV-1 capture efficiency. (B)** **HSV-2 capture efficiency. Download TABLE S1, PDF file, 0.1 MB. Copyright © 2018 Greninger et al. 2018 Greninger et al. This content is distributed under the terms of the Creative Commons Attribution 4.0 International license.

We found on average 10,000× enrichment of viral sequences with our capture panels with a maximum of 100,000× (Fig. 3). With this approach, we have recovered whole genomes from HSV-1/2 samples with viral loads lower than 10^2^ copies/reaction. Using an arbitrary cutoff of a 5% on-target fraction of postcapture HSV reads, we can recover genomes from precapture ratios of 10^−7.40^ for HSV-1 and 10^−5.78^ for HSV-2, corresponding to approximately 10^3^ copies/ml for HSV-1 and 10^4^ copies/ml for HSV-2. Based on these copy numbers, we calculate that with capture sequencing we will be able to recover whole HSV genomes directly from nearly all swabs obtained by our clinical lab for symptomatic lesions and approximately 85% of HSV-positive swabs from asymptomatic persons for clinical studies (26).

### Sequencing of culture versus clinical specimens in HSV-1.

With the ability to recover whole HSV genomes directly from clinical specimens, we sought to address to what extent sequence obtained from HSV-1 isolates obtained during routine culture in our clinical virology lab reflects viral sequence present in clinical swabs. We obtained 17 pairs of original clinical HSV-1 swabs in universal transport medium that had associated positive HSV-1 culture results on human fibroblast cells. These HSV-1 isolates were derived from a variety of specimens, including bronchoalveolar lavage, oral swabs, vaginal swabs, and penile swabs (Table S2). All HSV-1 isolates were in culture for fewer than 7 days (range of 2 to 7 days), and only one isolate (sample G9) was passaged after isolation.

10.1128/mSphereDirect.00283-18.4 TABLE S2  (A) HSV-1 swab and culture sequencing metadata. (B) History of HSV-1^+^ clinical samples in culture. Cytopathic effect was checked every day and graded on a scale of 0 to 4 before harvest (H). Download TABLE S2, PDF file, 0.1 MB. Copyright © 2018 Greninger et al. 2018 Greninger et al. This content is distributed under the terms of the Creative Commons Attribution 4.0 International license.

We sequenced these samples to a median of 547,494 reads (interquartile range [IQR], 352,038 to 830,777; *n* = 34), and we recovered near-full-length consensus genomes from as few as 101,000 reads. Median coverage was 518× (IQR, 276- to 741×; *n* = 34) with up to 99.6% of quality and adapter-trimmed reads being on target for HSV-1 (median, 99.2%; IQR, 99.0 to 99.3%; *n* = 34).

**FIG 4:**
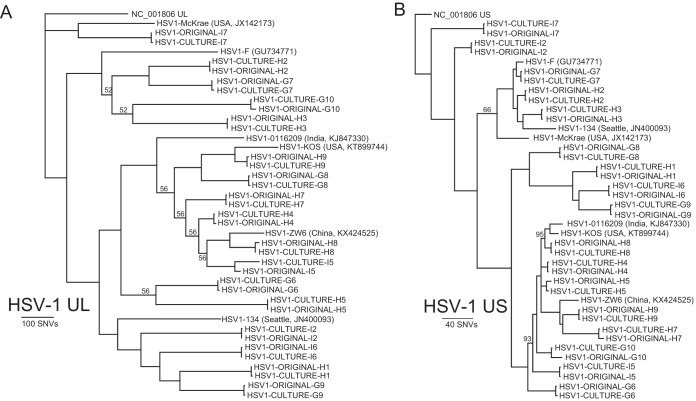
Limited evolution of HSV-1 during isolation in culture compared to sequence obtained directly from clinical samples. Phylogenetic analysis of UL (A) and US (B) sequences from HSV-1 subjected to capture sequencing after isolation in culture or directly from clinical sample. Across 14 of the paired samples, no single nucleotide variant was found in the UL or US region that was not present in homopolymers or UL36, US5, or US12 repeat regions. Of note, samples H4 and I5 were from the same patient 18 days apart, illustrating HSV-1 oral superinfection. The long tree branch on the I5 consensus sequence is due to changes in allele frequencies due to competitive viral growth *in vitro* between the superinfecting strains. All branch posterior probabilities are >99% unless otherwise noted.

HSV-1 unique long (UL) and unique short (US) sequences recovered directly from clinical specimens were nearly identical to those recovered after isolation from human fibroblasts (Fig. 4). Allowing for all mutations, UL-US culture pairs had on average 20 single nucleotide variants (SNVs) (range, 2 to 59), and most of these were present in repetitive elements in genes US12 and US5 that likely represent sequencing/assembly artifacts. After accounting for missing data (N’s); homopolymers (>8 nucleotides); and sequencing/assembly artifacts due to difficult loci such as high-GC repeats in UL36, US5, and US12 genes, 14 of the 17 pairs of specimens were entirely identical in the UL-US region. One verified mutation was recovered in sample pair H5, with a synonymous C→T mutation in the consensus sequence at nucleotide 603 in UL39. The original H5 sample had a 55% C, 45% T allele frequency at the locus, while the culture sample was 4% C, 96% T.

Sample G10 had four mutations between culture and clinical sample, including three synonymous changes in UL6, UL37, and UL54 and a T207A nonsynonymous mutation in the US7 coding sequence. All four mutations in G10 and the single mutation in H5 were confirmed by Sanger sequencing of the paired culture and original samples. The original sample for G10 had a notably low level of HSV-1 (18 copies/μl DNA or 9,000 copies/ml), and its assembly was 9.1% missing data (N’s). There was no evidence of HSV-2 recombination in the 17 pairs of HSV-1 sequences.

### Detection of HSV-1 superinfection.

Sample pairs H4 and I5 were collected from the same patient in his 50s who underwent two allogeneic hematopoietic cell transplants for acute myelogenous leukemia. The first sample (H4, “day 1”) was collected from a tongue ulcer, and the second sample (I5, “day 18”) was taken from an oral swab of a new tongue lesion 18 days later. He started foscarnet induction therapy 4 days prior to the first sample for treatment of cytomegalovirus (CMV) reactivation but was not treated with acyclovir in the intervening period. The day 1 oral swab measured 10^5.9^ copies/ml for HSV-1, while the day 18 oral swab measured 10^5.4^ copies/ml.

After removing SNVs associated with the UL36 gene, the consensus UL sequences recovered from the two original oral swabs differed by 207 nucleotides, which is consistent with previous estimates of average pairwise SNV differences between two unrelated HSV strains (6, 9). The consensus UL sequence from the day 1 original sample and culture specimen differed at only 3 nucleotides, which were all associated with homopolymers, consistent with the lack of evolution seen during culture isolation for 13 other paired HSV-1 specimens. However, the consensus UL sequence from the day 18 original sample and culture differed by 91 nucleotides, illustrating a rate of change significantly higher than that seen in other paired specimens.

**FIG 5:**
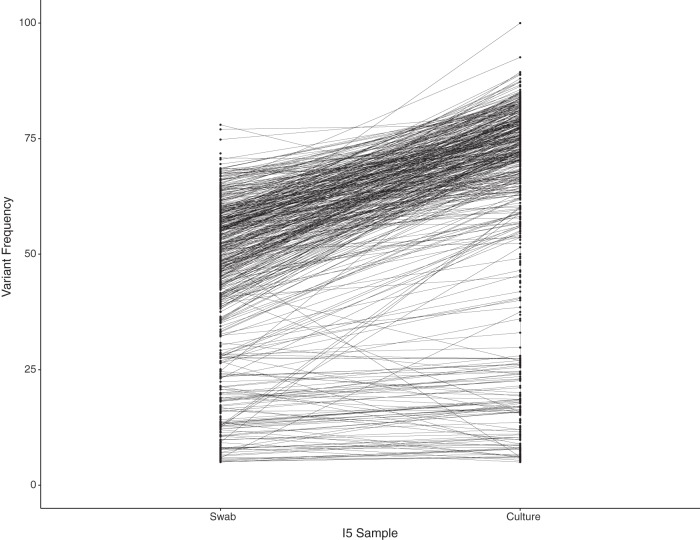
Allele frequency changes for the I5/“day 18” original oral swab HSV-1 genome and associated culture HSV-1 genome. The original consensus genome for the day 1 swab was used as a common reference from which to calculate allele frequency changes. The majority of alleles increase in frequency, crossing the 50% frequency threshold, resulting in artifactual evolution in culture that is the result of competition between mixed strains in culture.

We hypothesized that changes in variant frequency between two different viral populations present in the day 18 specimen accounted for the increased rate of change during isolation in culture. Mapping of the day 18 original sample and cultured virus reads to the consensus day 1 original sample complete genome revealed 609 and 620 single nucleotide variants, respectively, with minor allele frequency of >5% and depth of >25×. Most (92%) of the matched variant alleles increased in frequency from the original swab to the culture genome, from a median 45% to 66% allele frequency between the two specimens (Fig. 5). These data suggest that the differences in consensus genome between the culture and original day 18 specimens were due to allele frequency changes across the 50% consensus threshold within a mixed infection.

Since the patient’s HSV-1 emerged during foscarnet therapy, we next interrogated our sequence data for whether antiviral resistance was present in either of the oral swabs. Four nonsynonymous mutations were present in the UL30 gene from the day 18 oral swab compared to the day 1 oral swab at various allele frequencies (S724N, 6%; E798K, 11%; I810L, 16%; F918L, 57%). Compared to the HSV-1 strain 17 reference genome (NC_001806), both original samples had consensus UL30 coding changes at S33G, V905M, P920S, P1199Q, and T1208A. None of these changes has previously been reported to be associated with foscarnet resistance (27–29). These results are consistent with the patient being superinfected with two separate HSV-1 strains that reactivated at separate times on the patient’s tongue and were simultaneously detected from the day 18 specimen. These data also indicate that in the setting of superinfection, cultured samples may appear very different from swab samples due to differential abilities of the multiple viruses to grow in culture.

## DISCUSSION

We report the validation of capture sequencing panels for obtaining near-complete HSV-1 and HSV-2 genomes directly from clinical samples. The panels allow the recovery of HSV-1 and HSV-2 genomes in approximately 3 to 5 days with as few as 100,000 paired-end reads at viral concentrations that are up to 100,000-fold lower than those previously reported for herpes simplex viruses. The level of enrichment seen here is similar to that seen by others using capture panels (30, 31). We used this panel to show that HSV-1 undergoes extraordinarily limited evolution during culture isolation, finding only 5 single nucleotide variants across more than 1.8 Mb of UL-US sequence from 15 paired HSV-1-positive samples.

To date, direct-from-sample whole-genome sequencing for herpes simplex viruses has been limited to samples with extraordinarily high viral copy numbers (9). The vast majority of genome sequence data available from herpes simplex viruses comes from culture isolates. Our data indicate that these culture isolate sequences likely faithfully represent the original herpes simplex virus sequence present in the clinical samples from which the viral isolate originated.

Despite the success of culture in faithfully amplifying genomes, capture sequencing direct from patient samples has a number of advantages. Clinical samples at low HSV copy number often do not yield positive cultures. Even high-copy-number culture samples may consist of less than 1% HSV-1 reads, and thus, captured libraries can be sequenced in greater depth and in a more multiplexed fashion. Capture sequencing of HSV for genotypic antiviral resistance for drugs such as acyclovir or foscarnet may also return results faster than phenotypic culture-based tests, which require growth of the virus and have a relatively long turnaround time. Though this assay can be performed in as little as 72 h, we envision that a capture-based whole-genome genotypic clinical test for antiviral resistance or epidemiological purposes would likely be batched weekly with a sample-to-answer turnaround time of 5 to 11 days, depending on when the sample is received and the required test volume. Engineering and automation improvements to the protocol could substantially reduce hands-on time and lead to significantly shorter turnaround times.

We also use direct-from-sample sequencing to show the first case of HSV-1 superinfection detected directly from a patient by next-generation sequencing. The prevalence of HSV-1-infected individuals who carry more than one HSV-1 strain is not known, while HSV-2 superinfection is estimated to occur in approximately 3.5% of patients positive for HSV-2 (10). Despite HSV-1 reactivating in this patient in the setting of foscarnet treatment, no previously characterized mutations for foscarnet resistance were discovered (27, 29, 32). These data underscore the current challenge in confidently assigning antiviral resistance for HSV through genomic sequence.

Limitations of our study include examining HSV-1 evolution in the context of brief culture exposure with minimal passage. Our results may not be reflective of strains that undergo more passages than the initial viral isolation (“zero passage”) that was performed here. Notably, we and others have also not solved the problem of the high degree of homopolymers and repetitive sequence in the setting of high GC content in human herpes simplex viruses. Indeed, several of the loci cannot be confidently synthesized as oligonucleotides for the affinity purification panel. We also limited our sequence analysis to the UL and US regions of the genome.

In summary, we demonstrate the validation of a new robust, accurate, and sensitive tool to recover near-complete HSV-1 and HSV-2 genome sequences, along with an easy pooling scheme to reduce overall sequencing costs. We show that HSV-1 culture isolates undergo very few genomic changes in the UL-US region during isolation in culture. Indeed, culture may be the ultimate viral enrichment method for HSV-1 and HSV-2.

## MATERIALS AND METHODS

### Clinical samples.

HSV-1 and HSV-2 samples were selected from natural history research studies at the University of Washington Virology Clinic that spanned a range of precapture viral concentrations (see Table S1 in the supplemental material). Excess HSV-1 samples sent to the University of Washington Clinical Virology Lab for culture over a 1-month period in 2017 were also selected for sequencing (Table S2). Informed consent was obtained for HSV-1 and HSV-2 specimens from the Virology Clinic. Informed consent was waived for HSV-1 original swab and culture evolution samples by the University of Washington Human Subjects Division based on use of deidentified excess HSV-1 clinical specimens. The University of Washington Human Subjects Division approved both procedures.

### Swab DNA extraction and qPCR.

DNA was extracted from 200 µl of proteinase K buffer in which the original swab specimen was placed or from 40 µl of viral culture supernatants using the QIAamp DNA blood minikit (Qiagen). DNA was eluted into 100 µl of AE buffer provided in the extraction kit, and 10 µl of the DNA was then used for each real-time PCR. HSV DNA copy number was measured by an HSV-type common real-time PCR assay which amplifies the gB gene (33). Human genomic number in the original swab samples was measured by the primers and probe designed to detect the beta-globin gene (betaF, TGA AGG CTC ATG GCA AGA AA; probe, TCC AGG TGA GCC AGG CCA TCA CT; betaR, GCT CAC TCA GTG TGG CAA AGG). Each 30-µl PCR mixture contained 10 µl of purified DNA, 833 nM primers, 100 nM probe, internal control, and 15 µl of QuantiTect multiplex 2× PCR master mix. The thermocycling conditions were as following: 50°C for 2 min and 95°C for 15 min, followed by 45 cycles of 94°C for 1 min and 60°C for 1 min.

### PCR/Sanger sequencing.

PCRs of HSV-1 and HSV-2 UL23 genes and discrepant loci were performed using the PrimeSTAR GXL DNA polymerase (TaKaRa) with the primer sequences available in Table S3A and B. Each 50-µl PCR mixture contained 10 µl DNA, 10 µl 5× PrimeSTAR GXL buffer, 0.2 mM deoxynucleotide triphosphate (dNTP), 0.32 µM primers, and 1.25 units of PrimeSTAR GXL DNA polymerase. PCRs were performed using the following conditions: 98°C for 45 s; 40 cycles of 98°C for 10 s, 60°C for 15 s, and 68°C for 120 s; and 68°C for 10 min. Confirmatory PCR for discrepant loci was performed using the following conditions: 98°C for 415 s; 40 cycles of 98°C for 10 s, 55°C for 15 s, and 68°C for 30 s; and 68°C for 5 min. Sanger sequencing reactions were performed using the sequencing primers in Table S3C.

10.1128/mSphereDirect.00283-18.5 TABLE S3  (A) PCR primers for U23. (B) Primers for PCR and confirmatory Sanger sequencing of discrepant original swab versus culture samples. (C) Sequencing primers for U23. Download TABLE S3, PDF file, 0.02 MB. Copyright © 2018 Greninger et al. 2018 Greninger et al. This content is distributed under the terms of the Creative Commons Attribution 4.0 International license.

### Capture sequencing of HSV-1 and HSV-2 samples.

We first optimized the fragmentation and library preparation steps on high-concentration HSV-2 culture specimens, comparing Nextera XT, Kapa HyperPlus, and custom New England BioLabs (NEB) Fragmentase-based protocols. Kapa HyperPlus and NEB Fragmentase gave equivalent coefficients of variation for genome coverage (29.2% versus 31.0%, respectively), while the Nextera XT coefficient of variation was three times higher (96.7%), likely due to the known GC bias of the enzyme (data not shown). We subsequently chose to perform precapture library preparation using half-volumes of Kapa HyperPlus with a 7-min fragmentation step on 100 ng of DNA, ligation of 15 µM common Y-stub adapters, and 0.8× AMPure postligation cleanup. Postligation PCR amplification was performed using the Kapa HiFi HotStart ReadyMix with TruSeq dual-indexed primers (98°C for 45 s; 12 cycles of 98°C for 15 s, 58°C for 30 s, and 72°C for 30 s; and 72°C for 1 min) and cleaned using 0.8× AMPure beads. Precapture libraries were quantitated on a Qubit 3.0 fluorometer (Thermo Fisher).

Prior to capture, libraries were pooled in sets of 4 to 10 libraries based on the ratio of HSV-1/2 to beta-globin and total number of HSV-1/2 copies present in each library. A total of 300 to 500 ng DNA was targeted for each pool. We aimed for less than 10-fold variance from the highest to lowest concentration in HSV-1/2 copies within each pool. Hybridization capture was performed according to the IDT xGen protocol (version 2). Capture panels were designed as 1- by 120-bp tiling panels according to HSV-1 strain 17 and HSV-2 HG52 reference sequences (NC_001806 and NC_001798, respectively) with human masking based on IDT xGen design. Oligonucleotide capture panel sequences are available in Data Sets S1 and S2.

10.1128/mSphereDirect.00283-18.6 DATA SET S1  Capture panel design for HSV-1. Download DATA SET S1, XLSX file, 0.1 MB. Copyright © 2018 Greninger et al. 2018 Greninger et al. This content is distributed under the terms of the Creative Commons Attribution 4.0 International license.

10.1128/mSphereDirect.00283-18.7 DATA SET S2  Capture panel design for HSV-2. Download DATA SET S2, XLSX file, 0.1 MB. Copyright © 2018 Greninger et al. 2018 Greninger et al. This content is distributed under the terms of the Creative Commons Attribution 4.0 International license.

### Computational pipeline for assembly and annotation of HSV genomes.

Our workflow combines multiple open-source tools with custom shell and R scripts to rapidly extract and annotate near-full-length HSV genomes from raw Illumina sequencing reads (Fig. 2). All code is available on Github (https://github.com/proychou/HSV).

Raw sequencing reads (either paired or single end) in fastq format are trimmed to remove adapters and low-quality regions using BBDuk (34). Quality control (QC) reports are generated on the raw and preprocessed files using FastQC (35). Optionally, non-HSV reads are filtered out using BBDuk with *k* = 31 and hdist = 2. Preprocessed reads are *de novo* assembled using SPAdes, and contigs are ordered by aligning to HSV-1 or -2 reference sequences (NC_001806, NC_001798, and KF781518) using Mugsy (36, 37). A custom script in R/Bioconductor is used to fill in any gaps between contigs to create a template, and reads are mapped to this template using Bowtie 2 (38). A second script using R/Bioconductor is used to construct and clean up the final consensus sequence and prepare files for annotation. Annotation is performed using Prokka and a custom script to construct the final consensus sequence (39).

Although designed to be run on a high-performance computing (HPC) cluster, the code can also be run on a desktop computer. Additional wrapper scripts are available for parallelization of samples on an HPC cluster with scheduling systems like SLURM or PBS/Torque. Consensus sequences for each pair were aligned using MAFFT, pairwise differences calculated, UL-US sequences extracted, and locations of differences determined by adding annotations from HSV-1 references. The ggplot2 and nplr packages were used in R to calculate the limits of genome recovery (40). Phylogeny was created using MrBayes with default parameters (41).

### Recombination analysis of HSV-1 culture.

HSV-1 isolate sequences were examined individually for HSV-2 recombination using alignment trios with chimpanzee HSV (NC_023677.1) and an HSV-2 reference sequence (KF781518.1) as input for RDP (version beta 4.95). The RDP program was run from the command line with the default settings (42). This program uses the RDP, GENECONV, chimaera, and MaxChi algorithms to both detect events and verify events identified by other algorithms. The algorithms BootScan, SiScan, and 3Seq are computationally intensive when used to detect new events and so are used only to verify other events when using the default settings. All output files were combined and screened for *P* values of <1 × 10^−10^ for at least three algorithms. Results were the same when all putative events having a *P* value of 1 × 10^−10^ or smaller for only 2 algorithms were considered.

### Culture of HSV-1 isolates.

Swab samples were collected and transported to the clinical lab in universal transport medium. Supernatant fluid was removed, diluted with Hanks balanced salt solution (HBSS) with antibiotics, and centrifuged at 700 × *g* for 10 min, and 0.2 ml was inoculated into duplicate human fibroblasts (MRHF) (Diagnostic Hybrids). Cell monolayers were observed microscopically daily for HSV cytopathic effect (CPE). If typical CPE was noted, culture medium was harvested and frozen at −80°C for PCR analysis. To confirm the subtype of the isolate, MRHF cells were scraped and spotted onto slides with wells, air dried, fixed in acetone, and stained with monoclonal antibody to HSV-1 and HSV-2 (MicroTrak; Trinity Biotech).
