# Supplementary material for: Ultrasensitive Capture of Human Herpes Simplex Virus Genomes Directly from Clinical Samples Reveals Extraordinarily Limited Evolution in Cell Culture
Source: mSphere. 2018 Jun 13;3(3):e00283-18. doi: 10.1128/mSphereDirect.00283-18 (PMC6001610; doi:10.1128/mSphereDirect.00283-18)
Supplement: TABLE S1 [file sph003182571st1.pdf]

Table S1A. HSV-1 capture efficiency

Sample	HSV copies/mL	BG copies/mL	Precapture fraction	Post-capture %	SampleSite
2003-15756	2.04E+03	2.27E+05	4.50E-07	25.30	ORAL
2004-63623	1.00E+07	7.53E+04	6.68E-03	97.23	GEN L
2005-03043	4.76E+03	5.08E+06	4.68E-08	0.52	ORAL L
2005-51211	9.04E+07	5.62E+05	8.04E-03	88.03	ORAL L
2006-43275	9.33E+03	2.25E+06	2.07E-07	1.23	ORAL L
2006-57630	1.39E+05	1.68E+06	4.12E-06	8.59	ORAL
2006-57683	3.45E+08	7.25E+06	2.38E-03	97.73	GEN L
2007-01997	2.26E+05	1.98E+06	5.71E-06	23.63	ORAL
2007-03494	3.96E+04	7.24E+06	2.74E-07	7.21	ORAL
2007-12932	1.79E+05	2.71E+06	3.31E-06	41.30	ORAL
2007-16123	6.97E+03	4.40E+06	7.93E-08	12.02	ORAL
2007-16426	3.31E+05	3.45E+06	4.80E-06	34.57	ORAL
2007-21335	1.80E+04	1.30E+06	6.89E-07	12.51	ORAL
2007-21986	9.46E+03	1.18E+07	4.00E-08	0.18	ORAL
2009-19950	9.87E+03	8.13E+05	6.07E-07	12.01	ORAL
2009-20371	7.10E+05	2.17E+06	1.64E-05	93.80	ORAL L
2009-25015	1.19E+05	1.38E+06	4.29E-06	63.74	ORAL
2009-25575	1.51E+04	3.90E+06	1.94E-07	9.01	ORAL
2009-25772	5.72E+04	6.26E+05	4.57E-06	70.05	ORAL
2009-29406	8.94E+03	3.83E+06	1.17E-07	9.62	ORAL
2009-29964	8.59E+04	5.70E+05	7.54E-06	90.03	ORAL
2009-39273	3.28E+05	1.73E+06	9.48E-06	98.35	ORAL
2009-39486	1.96E+04	2.87E+06	3.41E-07	15.93	ORAL
2010-21371	8.17E+04	2.22E+06	1.84E-06	44.43	ORAL
2010-25244	4.01E+04	2.84E+06	7.06E-07	35.70	ORAL
2010-28641	4.34E+04	1.44E+06	1.50E-06	31.16	ORAL
2010-28683	3.92E+05	3.39E+06	5.79E-06	79.99	ORAL
2010-32082	3.78E+05	5.27E+06	3.59E-06	81.21	ORAL
2011-1026	3.83E+05	2.09E+06	9.17E-06	89.46	ORAL
2011-12689	1.02E+08	4.63E+05	1.10E-02	96.84	GEN L
2011-12714	3.88E+06	1.32E+05	1.47E-03	97.00	GEN L
2011-12715	6.94E+07	1.85E+04	1.88E-01	99.34	GEN L
2011-12717	1.73E+08	8.43E+05	1.03E-02	97.62	GEN L
2011-12719	2.36E+07	2.02E+05	5.85E-03	97.43	GEN L
2011-12741	1.69E+06	5.58E+04	1.52E-03	96.55	GEN L
2011-12742	2.91E+06	5.74E+04	2.53E-03	96.80	GEN L
2011-13906	1.84E+03	2.09E+06	4.39E-08	23.10	ORAL
2011-15240	4.06E+03	1.83E+06	1.11E-07	69.20	ORAL
2011-15562	8.68E+03	6.42E+05	6.76E-07	2.85	ORAL
2011-16318	5.06E+05	1.27E+07	2.00E-06	4.09	ORAL

2011-17275	1.36E+06	3.10E+06	2.20E-05	84.69	ORAL
2011-21041	8.08E+07	3.09E+06	1.31E-03	98.52	ORAL
2011-31164	4.91E+06	5.23E+06	4.70E-05	97.05	ORAL L
2011-3153	1.22E+05	8.99E+06	6.77E-07	19.51	ORAL
2011-5409	6.40E+06	2.70E+05	1.19E-03	99.18	ORAL L
2011-5438	5.74E+03	4.29E+06	6.69E-08	35.53	ORAL
2014-32338	1.51E+08	2.14E+06	3.52E-03	97.48	GEN L
2015-30637	1.26E+08	1.29E+05	4.91E-02	98.07	GEN L
2016-01040	6.97E+07	1.22E+06	2.86E-03	95.97	ORAL

Table S1B. HSV-2 capture efficiency

Sample	HSV copies/mL	BG copies/mL	Precapture fraction	Post-capture %	SampleSite
2007-675	8.02E+06	9.74E+04	4.12E-03	99.19	LES
2008-14582	6.54E+08	1.00E+07	3.26E-03	98.49	MIX
2009-34871	9.40E+04	8.42E+04	5.58E-05	62.96	MIX
2010-1864	1.09E+08	2.91E+05	1.87E-02	99.49	VULV
2010-28267	4.27E+07	1.13E+06	1.89E-03	97.37	GEN
2012-15945	1.60E+06	1.80E+06	4.44E-05	92.94	MIX
2012-26413	1.30E+07	9.02E+05	7.20E-04	95.65	MIX
2012-30563	1.12E+06	5.60E+06	1.00E-05	72.10	LES
2013-16478	9.40E+08	3.04E+06	1.54E-02	98.08	LES
2013-30421	3.86E+07	9.17E+04	2.10E-02	99.50	LES
2013-36269	5.40E+07	9.59E+05	2.82E-03	99.19	LES
2013-36329	1.22E+06	1.47E+06	4.15E-05	77.91	MIX
2013-41805	7.92E+06	2.71E+06	1.46E-04	90.59	MIX
2014-15985	9.56E+07	1.05E+06	4.57E-03	98.51	LES
2014-18610	4.57E+06	4.46E+06	5.12E-05	85.13	MIX
2014-19576	1.46E+07	2.85E+04	2.55E-02	99.45	LES
2014-21348	1.18E+05	3.78E+06	1.57E-06	2.83	MIX
2014-23182	6.79E+06	2.29E+06	1.48E-04	94.82	MIX
2014-23866	1.43E+06	3.47E+05	2.05E-04	43.59	LES
2015-11127	1.78E+07	3.07E+05	2.90E-03	99.40	LES
2015-11486	5.99E+05	4.63E+06	6.46E-06	35.72	MIX
2015-14667	5.27E+05	7.14E+05	3.69E-05	66.44	MIX
2015-16311	1.42E+06	5.24E+05	1.36E-04	94.81	MIX
2015-16590	1.40E+05	1.20E+06	5.86E-06	36.20	LES
2015-19852	6.18E+04	4.17E+03	7.41E-04	99.10	LES
2015-20449	3.93E+07	3.82E+05	5.14E-03	99.25	MIX
2015-25717	7.60E+06	3.25E+06	1.17E-04	90.99	MIX
2015-25722	9.11E+04	3.13E+06	1.46E-06	3.98	MIX
2015-25744	2.70E+07	6.28E+06	2.15E-04	92.32	MIX
2015-29174	1.77E+04	3.85E+05	2.29E-06	6.01	MIX
2015-29818	5.27E+08	3.01E+06	8.74E-03	98.90	MIX
2015-30634	2.71E+06	5.44E+05	2.50E-04	97.43	LES
2015-4464	1.57E+07	5.66E+05	1.39E-03	99.01	LES
2016-11170	1.05E+07	6.91E+05	7.62E-04	98.48	MIX
2016-13079	7.72E+05	7.31E+04	5.28E-04	96.65	MIX
2016-1753	8.49E+05	1.19E+05	3.58E-04	98.04	LES
2016-833	4.85E+03	1.59E+06	1.53E-07	0.87	MIX
