# Supplementary material for: Ultrasensitive Capture of Human Herpes Simplex Virus Genomes Directly from Clinical Samples Reveals Extraordinarily Limited Evolution in Cell Culture
Source: mSphere. 2018 Jun 13;3(3):e00283-18. doi: 10.1128/mSphereDirect.00283-18 (PMC6001610; doi:10.1128/mSphereDirect.00283-18)
Supplement: TABLE S2 [file sph003182571st2.pdf]

Table S2A. HSV-1 swab and culture sequencing metadata

Sample ID	raw_reads	reads_postproccess	mapped_hsv1	%mapped	Average_HSV1_coverage	percent_Ns	Accession
HSV1-CULTURE-G10	1229412	1097610	1090114	99.32	1208.81	4.53	MH160375
HSV1-ORIGINAL-G10	1035958	967430	949861	98.18	901.13	4.39	MH160388
HSV1-CULTURE-G6	221264	202132	198673	98.29	190.37	3.84	MH160389
HSV1-ORIGINAL-G6	581972	518272	514028	99.18	537.42	3.46	MH160390
HSV1-CULTURE-G7	645988	587056	582151	99.16	634.67	4.41	MH160382
HSV1-ORIGINAL-G7	423612	375366	372124	99.14	408.50	6.16	MH160361
HSV1-CULTURE-G8	2864832	2546146	2527820	99.28	2596.75	1.57	MH160386
HSV1-ORIGINAL-G8	472060	424672	420931	99.12	453.70	3.81	MH160383
HSV1-CULTURE-G9	776544	701670	698603	99.56	683.97	2.80	MH160369
HSV1-ORIGINAL-G9	652408	584640	581140	99.40	628.88	3.38	MH160379
HSV1-CULTURE-H1	1621614	1400298	1388433	99.15	1476.37	2.55	MH160362
HSV1-ORIGINAL-H1	1000342	883656	877773	99.33	980.71	4.62	MH160367
HSV1-CULTURE-H2	1092846	960480	953645	99.29	1042.15	2.62	MH160385
HSV1-ORIGINAL-H2	351962	307350	304509	99.08	338.40	5.21	MH160377
HSV1-CULTURE-H3	525172	474634	471038	99.24	513.88	4.22	MH160365
HSV1-ORIGINAL-H3	230368	206674	204975	99.18	217.67	5.18	MH160359
HSV1-CULTURE-H4	595994	540544	537450	99.43	544.02	2.37	MH160381
HSV1-ORIGINAL-H4	236910	216666	215233	99.34	235.01	5.98	MH160374
HSV1-CULTURE-H5	677514	600190	597023	99.47	668.28	4.13	MH160358
HSV1-ORIGINAL-H5	547494	488780	486660	99.57	517.60	4.81	MH160373
HSV1-CULTURE-H7	1086048	960728	951629	99.05	1056.20	3.12	MH160376
HSV1-ORIGINAL-H7	261148	241390	231235	95.79	255.74	5.95	MH160363
HSV1-CULTURE-H8	1434402	1321826	1314595	99.45	1373.32	2.92	MH160391
HSV1-ORIGINAL-H8	481492	426000	423326	99.37	465.21	4.21	MH160364
HSV1-CULTURE-H9	624514	554094	549392	99.15	604.29	5.32	MH160368
HSV1-ORIGINAL-H9	126758	115684	114437	98.92	127.93	7.54	MH160378
HSV1-CULTURE-I2	737344	652112	648297	99.41	703.07	3.57	MH160380
HSV1-ORIGINAL-I2	418622	380952	378106	99.25	416.02	5.34	MH160370
HSV1-CULTURE-I5	186844	165492	163782	98.97	175.33	3.96	MH160372
HSV1-ORIGINAL-I5	355696	318162	315861	99.28	336.78	2.95	MH160384
HSV1-CULTURE-I6	848854	759178	748867	98.64	753.10	2.37	MH160360
HSV1-ORIGINAL-I6	352268	330920	191702	57.93	169.62	5.35	MH160371
HSV1-CULTURE-I7	111036	95212	92282	96.92	103.30	8.72	MH160387
HSV1-ORIGINAL-I7	101166	92752	88318	95.22	88.75	7.51	MH160366

Supplemental Table 2B - History HSV1+ clinical samples in culture. Cytopathic effect was checked every day and graded on a scale of 0-4 before harvest. H=Harvest

Isolate	sample type	day 1	day 2	day 3	day 4	day 5	day 6	day 7	day 8	day 9	day 10
G6	oral swab	+/-	++	H							
G7	swab	-	++	H							
G8	vaginal ulcer	+/-	++	H							
G9	lip lesion	++++	++++/pass	+++	H						
G10	BAL	-	-	+/-	+	+	+++	H			
H1	pustule	-	+	++	++	H					
H2	vaginal ulcer	-	+	+++/H							
H3	SVTM	+/-	++	H							
H4	tongue ulcer	-	+	++/H							
H5	superficial le	++	++/H								
H7	SVTM	-	+	++	++	H					
H8	lesion	-	+	++++	++++/H						
H9	BAL RML	-	+/-	+++	++++/H						
I2	penile swab	-	+/-	+++	++++	H					
I5	oral swab	-	+/-	+++/H							
I6	throat	-	+/-	++	++	H					
I7	vulva/cervix	+	+++	H							
