# Supplementary material for: Ultrasensitive Capture of Human Herpes Simplex Virus Genomes Directly from Clinical Samples Reveals Extraordinarily Limited Evolution in Cell Culture
Source: mSphere. 2018 Jun 13;3(3):e00283-18. doi: 10.1128/mSphereDirect.00283-18 (PMC6001610; doi:10.1128/mSphereDirect.00283-18)
Supplement: TABLE S3 [file sph003182571st3.pdf]

Table S3A – PCR primers for U23

**HSV-2 TK**	
HSV2TKFwd46783	ACACACCACACGACAACAATG
HSV2TKRev48152	CGAGGTCCACTTCGCATATT
**HSV-1 TK**	
HSV1TKFwd46537	AACACCCGTGCGTTTTATTC
HSV1TKRev47880	ATATTAAGGTGACGCGTGTGG

Table S3B – Primers for PCR and confirmatory Sanger sequencing of discrepant original swab versus culture samples

G10_UL6-F	GAC ATT AAC GAC ACT GTG CG
G10_UL6-R	CCG TTG ATG TTG TTA ACC ACG G
G10_UL37-F	ACG CGC GCC ATT AGC GTC
G10_UL37-R	CAG CGC ATA GAC GAC GCG AC
G10_UL54-F	GCC GCG ACG ACC TGG AAT C
G10_UL54-R	AGG CGC GAC CAC ACA CTG TG
G10_US5-F	GAA GGT ACT CTG GCG TAC AAC
G10_US5-R	CAA GCA TCG ACC ACA CCC TTC
H5_UL39-F	CTC CTG TCG CGA CAC ACG
H5_UL39-R	AAT CGG AGT CAA GGG CGT CGT C

Table S3C – Sequencing primers for U23

HSV-1 TK gene:	
HSV1TKFwd46537	AACACCCGTGCGTTTTATTC
HSV1TKRev47800	ATCTTGGTGGCGTGAAACTC
HSV1TKFwd47039	GAATCGCGGCCAACATAG
HSV1TKFwd47337	GTCGGTCACGGCATAAGG
HSV1TKRev47123	CTTCCGGAGGACAGACACA
HSV1TKRev46907	CGTGCCGCCCCAGGGTGCCGAGC
HSV1TKFwd46828	GTTATACAGGTCGCCGTTGG
HSV1TKRev47535	GCAGAAAATGCCCACGCTACTG
HSV1TKFwd47478	GCCAGTAAGTCATCGGCTCGGGT
HSV-2 TK gene	
HSV2TKFwd46815	GTTCTTTTATTGCCGTCATCG
HSV2TKRev48067	CGTTCCACAAATCCTGGTG
HSV2TKFwd47279	ATCGTAGACACGGCGAATG
HSV2TKRev47297	CATTCGCCGTGTCTACGAT
HSV2TKFwd47635	CTGGTCATTACCACCGCCGCCTC
HSV2TKFwd47066	AAGACCCAGGCAAAAATGTGG
HSV2TKRev47135	CCCGCATCGAGGACACGCTGTT
